# Plastinate Library: A Tool to Support Veterinary Anatomy Learning

**DOI:** 10.3390/ani14020223

**Published:** 2024-01-10

**Authors:** Rafael Senos

**Affiliations:** 1 Department of Biomedical Sciences, College of Veterinary Medicine, Cornell University, 240 Farrier Road, Ithaca, NY 14853, USA; rafaelsenos@yahoo.com.br; 2 Department of Comparative Pathobiology, Cummings School of Veterinary Medicine, Tufts University, 200 Westboro Road, North Grafton, MA 01536, USA

**Keywords:** animal, education, morphology, plastination, remote, teaching

## Abstract

**Simple Summary:**

The challenges to learn veterinary anatomy in the actual curriculums and the changes in studying habits promoted by the global COVID-19 lockdown requires new approaches to deliver the best knowledge to the actual generation of learners. The present study evaluated the students’ acceptance to a educational approach, which consisted in bringing plastinate anatomical specimens to study at home. The assessment was done through an anonymous survey and individual interviews. Accordingly, to the students’ response, the method was well accepted for reasons that includes flexible time study and better possibilities to explore the anatomical differences between the species in three-dimensional materials. Considering the results and the low impact of this educational approach in the curriculum dynamics, the plastinate library is suggested as interesting aid to the learning experience in animal anatomy.

**Abstract:**

The shortage of both time for anatomy courses in the new veterinary medicine curriculum and instructors prepared to teach biomedical sciences has raised a crisis in anatomical education. Often, students spend time out of their classes trying to learn not only concepts but also laboratory-wise content from 2D materials such as books and videos. In addition, since the global COVID-19 pandemic lockdown, studying and habits have been reviewed, with many people adopting an at-home style. The purpose of this study was to evaluate students’ acceptance of taking plastinate anatomical specimens to study at home. Thirty-three students were divided into three groups. G1 took home a set of kidneys composed of equine, bovine, and swine materials; G2 took home a pig kidney; and G3 (control) did not take any plastinate specimen home. Acceptance was assessed using an anonymous survey and interview. The method had high acceptance by the students, who believed that having the plastinate library was advantageous from different perspectives, including aiding with learning the differences between kidneys from different species, time flexibility, no commute to study after hours of laboratory classes, and time redistribution to prioritize the laboratory topics. The plastinate library has the potential to be a supportive tool for anatomy students in the contemporaneous veterinary curriculum paradigm, considering that the G1 and G2 groups used the plastinate specimens at home to complement the time they spent in the laboratory.

## 1. Introduction

Anatomy of domestic animals is one of the most challenging courses/subjects in veterinary medicine programs. Complaints about traditional teaching/learning methods are common [1]. In addition, there is a global trend of reducing time allocated to gross anatomy courses, a lack of experienced instructors to teach anatomy as well as other basic biomedical sciences, and a decrease in investments in teaching anatomy in the laboratory [2,3,4,5,6].

Several attempts have been made to improve anatomy teaching within this new paradigm [5,7,8]. For example, plastination, 3D-computed tomography images, problem-based learning, bodypainting sessions, e-learning, videos, and alternative and supportive educational tools have all been implemented to suit the current demands [1,6,9,10,11,12,13]. Since each institution has specific teaching resources and curriculum policies, this can often interfere with new educational strategies; therefore, the introduction of new approaches is often a challenge for educators. Furthermore, new approaches are rarely tested, hence introducing bad practices due to misconceptions is not unusual.

**Figure 1:**
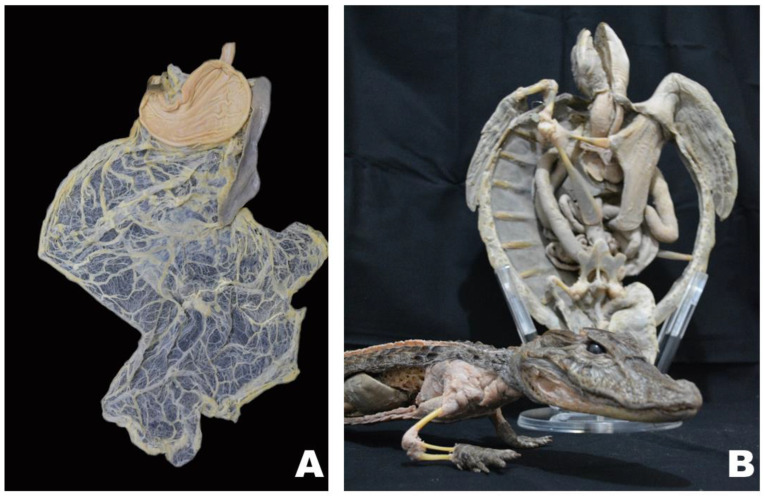
Plastinate veterinary specimens used for different purposes: (**A**) cat stomach, spleen, and greater and lesser omentum for teaching anatomy to DVM students; (**B**) Green turtle and Broad-snouted caiman specimens for museum presentations.

The plastination technique was first referred to by von Hagens and Knebel in 1978. This approach aims to replace the water and lipids in biological tissues with polymers. These polymers are then hardened, resulting in dry, odorless, and very durable preserved specimens. These characteristics have been appreciated by both the lay public in museum-type exhibitions and educators as a tool for teaching anatomy and pathology [14,15] (Sora et al., 2019; von Horst et al., 2019). (Figure 1).

Didactic plastination aims to produce specimens for educational purposes in universities, biological institutes, and museums. Clean, touchable, authentic, non-toxic, odor-free, and non-biohazardous specimens give the students a great advantage in manipulating real organs and body parts compared with classic medical and veterinary anatomical learning, which mostly relies on teaching using cadaveric materials fixed with hazardous chemicals [14]. One of the most remarkable advantages of plastinate specimens is the possibility of being handled outside the teaching laboratory without gloves.

Books and digital materials do not deliver content in three dimensions; thus, it is difficult for learners to understand complex topography outside of the laboratory, for example, from home [10,16]. In addition, during the COVID-19 lockdown, distance learning methods urged tested approaches to veterinary medical sciences [17,18,19]. Although it is not known whether this scenario will be repeated in the future, remote education is expanding rapidly and therefore the need to provide students with resources that they can access out of the laboratory may lead to a new perspective of using plastinate specimens to study at home.

Studying anatomy from home using real specimens has been reported to have good acceptance [19] in veterinary medical education. However, bringing specimens conserved using hazardous liquid chemicals home is controversial and probably not safe [20,21].

Based on the evidence, we aimed to give students access to plastinate specimens to study at home with the purpose of evaluating the acceptance of the method. We hypothesized that this approach could benefit some students in addition to the classic learning/teaching curriculum methods.

## 2. Methods: The Educational Experience

### 2.1. Ethical Considerations

This project was approved by the Social, Behavioral, and Educational Research Institutional Review Board of Tufts University. All the participants volunteered for the study by signing an informed consent. The participants could opt to withdraw from participation at any time and the data obtained from their answers would be automatically removed from the study. Participant animosity was respected throughout the study.

### 2.2. Context and Groups

The Doctor of Veterinary Medicine (DVM) program curriculum offered by the Cummings School of Veterinary Medicine includes two anatomy courses in the first year of the program. Anatomy 1 is a dissection-based course for the neck, thorax, abdomen, pelvis, and limbs of dogs and cats, which is taught over 106 h—60 h of laboratory work and 46 h of lectures during the Fall semester. The Anatomy 2 course is divided into two sections and is taught during the Spring: (A) a dissection-based course of dogs’ and cats’ heads taught for a total of 36 h—18 h of lectures plus 18 h of laboratory work—and (B) prosection-based course of large animal species taught over 72 h—28 h of laboratory work plus 44 h of lectures. The large animal section includes equine, bovine, caprine, ovine, swine, and South American camelids. The large animal laboratory classes consist of different stations with different organs in addition to dissected full-body specimens of the respective species. Each station consists of guiding material generated from pictures of the actual specimens, tag-key lists, and/or classic book materials, based on the Nomina Anatomica Veterinaria terminology [22,23,24,25,26,27].

The instructors move from station to station answering questions from the students during the laboratory classes. Students are free to choose which stations they want to access at any given time and how much time they want to study the stations during the laboratory classes.

Thirty-three students in the first year of the DVM program who were taking the Anatomy 2 course volunteered to participate in the study. To create the groups, each student was given a serial identification number; the students’ identification numbers were then randomly assigned to each of the three groups using a group-forming website (https://pt.rakko.tools/tools/59/, accessed on 18 March 2023). This way, each of the groups was composed of 11 students: (G1) Each student in this group took home a set of plastinate kidneys, including a bovine kidney, a pig kidney, and a pair (left and right) of equine kidneys connected by the renal arteries and the aorta; (G2) Each student in this group took home a single plastinate pig kidney; (G3) Control group, these students did not take any plastinate kidneys home.

Group G3 was established to contrast the interview answers with G1 and G2, especially to determine how beneficial the approach and the improvements in the anatomical description of the kidneys are.

**Figure 2:**
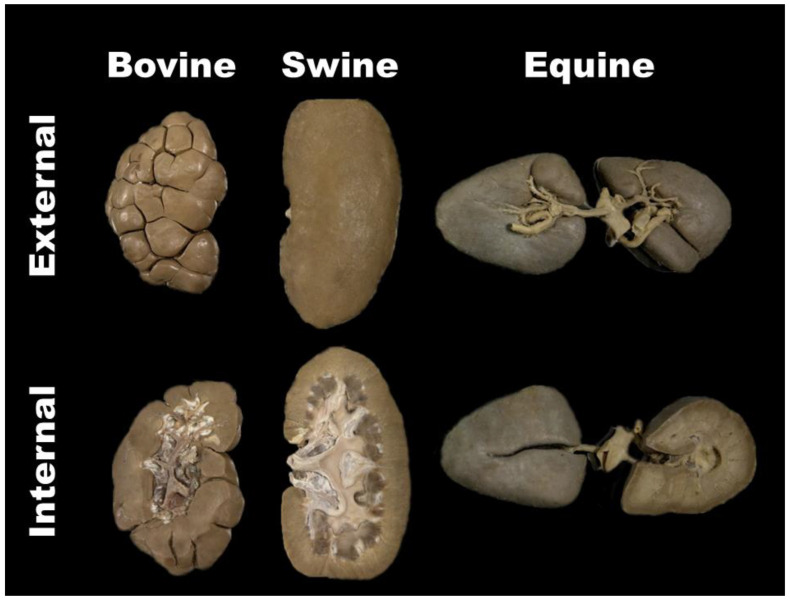
Representative specimens of the plastinate kidneys that the students in groups G1 and G2 took home. External and internal view in coronal plane section.

The bovine, pig, and left equine kidneys were sectioned in the coronal plane of the body at the level of the ureter (Figure 2).

### 2.3. Experimental Design

All the participants in the study, irrespective of group, had regular lectures and laboratory classes according to the normal curriculum. All the participants had access to plastinate kidneys (similar copies of the G1 set) in the laboratory environment based on the 24 h/7 days a week availability system.

Six days before the exam of abdominal and pelvic cavities of all large domestic animal species—which included the bovine, equine, and swine kidneys—G1 and G2 took the plastinate kidneys home with the instruction to not share them with the other participants in the study. This ensured that all the students had access to the kidneys with similar specimens in the laboratory environment. The only difference between the experimental groups (G1 and G2) and the control group (G3) was the opportunity to bring the kidney specimens home to study.

After the exam, all the kidneys that the students took home were returned to the school’s plastinate collection.

Qualitative and quantitative analyses were conducted to evaluate the students’ acceptance of this didactic approach.

### 2.4. Quantitative Acceptance: Method Acceptance Survey

To assess the students’ acceptance of the tested approach, students in the G1 and G2 groups could volunteer to answer an anonymous digital survey through the university’s official platform, Qualtrics (https://www.qualtrics.com).

The questions in the survey were objective and based on the 5-point Likert scale; there was also a question that the students answered using a percentage. The survey aimed to quantify data on the student’s interest in the didactic approach, the kind of learning that was obtained from the material, and whether the plastinate specimens were used at home to add study time to the routine or whether they were used to skip/replace the regular laboratory class time.

Statistics were generated using percentages and mean values.

### 2.5. Qualitative Assessment: Interviews

Although surveys are important for producing essential data, these evaluations have limitations and are frequently supplemented with qualitative data [28,29].

In addition to the exam results and the acceptance survey, three student volunteers from each of the G1, G2, and G3 groups were randomly selected for an interview to provide qualitative data to add to the study and support the quantitative data interpretation.

The interviewees were assured that they were not being tested and that no specific answer was expected. The interviews were conducted when the semester was over, therefore, no grades were jeopardized, and the author/professor of the study had no power over the students.

The interview questions were associated with the general practices in the anatomy laboratory, the preferences with respect to dissection-based versus prosection-based courses, and wet formalin-fixed specimens versus plastinate specimens. The interviewees were also invited to express how they felt about bringing the kidney sets (G1), the pig set (G2), or no kidneys (G3) home. Students in the G1 and G2 groups were also asked about the advantages and disadvantages of bringing the plastinate kidneys home. In the final part of the interview, all interviewees were asked to describe a kidney and compare it between species as if they were explaining it to a layperson.

## 3. Results

### 3.1. Quantitative Acceptance: Method Acceptance Survey

A total of 7 students in the G1 group and 11 students in the G2 group voluntarily answered the five questions on method acceptance in the anonymous online survey.

Students in the G1 group spent on average 1.7 h per person studying kidneys in the laboratory environment, while students in the G2 group spent 1 h. On the time spent studying the plastinate kidneys at home, students in the G1 group spent on average 1.4 h per person, while students in the G2 group spent 1.2 h.

**Figure 3:**
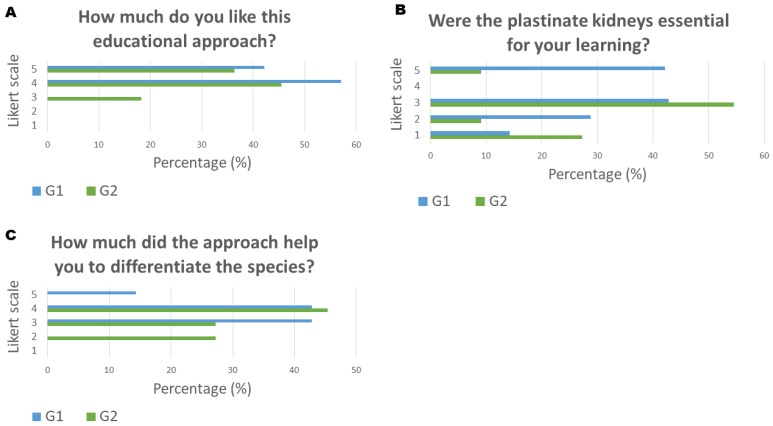
Results of plastinate library approach acceptance based on anonymous volunteer survey of 5-point Likert scale questions (1 min and 5 max) applied to the groups that took plastinate materials home. Group G1 took a plastinate set consisting of a bovine, a pig, and a pair of equine kidneys home; group G2 took a single plastinate pig kidney home.

When asked how much the students liked the approach, 16 of 18 volunteers (89%) ranked the approach as liking it somewhat or liking it a great deal. The G1 group ranked slightly higher than the G2 group for this specific question (Figure 3A).

Regarding how much the approach was essential to learning the anatomy of the kidney, the most frequent answer in both groups was a moderate amount. There were students who considered it not essential at all and others who answered that it was essential a great deal (Figure 3B).

The last part of the survey indicated that most of the students in group G2 considered the approach helpful a moderate amount or a lot in differentiating the kidneys by species, while 57% of students in group G1 answered a lot and a great deal to the same question. No student considered that bringing the plastinate material home did not help them to anatomically differentiate the kidneys by species (Figure 3C).

### 3.2. Qualitative Assessment: Interviews

During the interview, one student each from the G1, G2, and G3 groups answered that they missed the dissection practice for learning the anatomy of large animals, while the other six students said they preferred studying anatomy using prosected specimens because it is easier to learn when the structures/features were well dissected and evident. Two students suggested that a combination of using prosected specimens first and performing dissection next would be ideal.

On the comparison between wet/embalmed specimens and plastinate specimens, the students highlighted that the plastinate specimens were usually in better condition, with the structures presented more clearly, and were easier to manipulate. As for the wet specimens, one student from the G1 and the G2 groups pointed out that the wet/embalmed specimens helped them to understand the structures in different planes due to the mobility of the tissues and organs over the semester. All the students in all the groups indicated that they preferred studying plastinate prosected specimens over wet/embalmed prosected specimens.

All the students in all the groups indicated that they did not have enough time to cover all the material in the course during the regular laboratory classes. All the students indicated that studying in the laboratory environment after hours was necessary to complete their studies. One student in the G1 group and two students in the G3 group indicated that they preferred spreading their studies over short sessions after hours instead of studying more intensively during the laboratory classes because it was a better way of absorbing the content.

When asked whether taking plastinate kidneys home would improve their performance in the exam, all students in group G3, two students in group G2, and two students in group G1 answered positively. The two students who answered negatively to this question explained that their learning preference is to study inside the laboratory environment along with their classmates and that the kidney was a small topic among many others, hence they were not focused on that topic as they had also learned all the kidney principles during the anatomy 1 course in the previous semester.

Two students in group G1 and all students in group G2 felt that they had an advantage over their classmates because they took the kidneys home. According to these students, they had the opportunity to learn the kidney anatomy better and they could also use more time to study other materials in the laboratory. All students in group G3 felt that they had a disadvantage with respect to students in the G1 and G2 groups for not taking the kidneys home. All students in groups G1 and G2 mentioned that having the possibility of taking the kidneys home as in a library would give them more comfort and flexibility, especially for those who had to commute far to reach the laboratory after hours. No student reported any disadvantage to taking the kidneys home when they were asked specifically about it.

In the last part of the interview, students in the different groups described the morphology of the kidneys similarly in terms of details and analogies, for example, bean-shaped for a general description, and differences between the species, including a bunch of grapes-shaped for the bovine.

## 4. Discussion

Regarding the time management required to learn kidney anatomy, students in both G1 and G2 groups spent more time, on average, studying in the laboratory than at home. Nevertheless, considering both the students’ perception of their overall time management and the worldwide tendency to shorten anatomy curriculums [30,31,32,33], offering different resources to learn 3D anatomy out of laboratory classes may be a valuable resource for the current generation of learners. In this sense, the plastinate library emerges as a possible aid for some students.

Even though learning from home has advantages, being present in person in the laboratory seems to still be essential for learning anatomy for many students, based on our results and previous reports [17,34,35].

Although the majority of the answers indicated that bringing kidneys home had a moderate relevance to learning, there were students in both groups who indicated that the method was a big deal to learning kidney anatomy. The current generation of learners is more aware of their learning preferences compared with previous generations who were educated with fewer options to choose from [36,37]. During the interview, some students reported that they preferred learning alongside their peers in the laboratory; thus, having the specimens at home was not that important. On the other hand, having different materials and approaches to support student learning at home may help students with different learning styles [36,37,38,39]. In this case, it seems the plastinate library benefited the students who preferred to sometimes study away from the laboratory environment.

In an interesting trend in the survey answers, the majority of students in group G1 indicated that the kidney set helped them to distinguish differences between the species in comparison to students in the G2 group who only had pig kidneys and did not indicate having the kidney at home so positively. Learning the anatomy of different species is a key point in the large animals course taught at Tufts University and a major challenge for the students.

The perception the students had during the interview, that having access to the kidneys could give students an advantage, is interesting. The facilitated access to the kidneys allowed the students to distribute their time between home and laboratory. For example, the 1.2–1.4 h used to study kidneys at home may have freed a certain amount of time for the students to spend on other topics in the course and the laboratory. In addition, according to the students, the plastinate library could be of benefit to the students who have long commutes, resulting in a better use of time and resources and reduced stress for some specific students [40,41,42,43,44]. The high rank of how much the students liked the method, almost ninety percent of the volunteers in both groups G1 and G2, contrasts with no student ranking the approach below the middle. This may result in a potential tool for enhancing a curriculum that has been shortened in anatomy courses all over the world.

Although some advantages of the plastinate library were pointed out in this manuscript, the study had limitations such as a small sample size and the number of different organs used to test the approach. It is also necessary to comment that large organs, for example, large ruminants’ rumen or limbs, may not be suitable for this approach. Future studies should consider the efficacy of the method by testing the students’ learning using the plastinate specimens that were taken home compared with other topics that were not covered by the library.

## 5. Conclusions

Due to the multiplicity and variety of approaches available throughout the educational life of a contemporary DVM student, and all the different technologies and tools available, students have the possibility to almost customize their learning style, which may result in varying opinions on a specific approach. Nevertheless, this study presented evidence of specific learning benefits of the plastinate library to some individuals during the learning process, as most of the students enjoyed the approach, and found it helpful for learning the anatomy of the kidney and very helpful for differentiating the anatomical features of large domestic animal species. Furthermore, the results of this study also concluded that, according to the students, other advantages such as time flexibility, reduced costs associated with commuting, and flexible studying plans were important positive points in the global educational program. The plastination library showed an interesting potential to be a supportive method for learning veterinary anatomy because of the students’ acceptance of the method, which may compensate partially for the reduction in proper time required for learning anatomy during the time allocated to laboratory classes in the new curriculums and the lack of three-dimensional materials to review from home.

## Data Availability

Due to ethical principles, the data generated in this study is not available for the general public.
